# Diseases of the Liver

**Published:** 1905-12-16

**Authors:** 


### DISEASES OF THE LIVER.

**Cirrhosis.**—Notwithstanding the fact that cases of cirrhosis are fairly common, there is still much dispute as to the causation and pathology of this condition. In a recent clinical lecture Saundby [1] reports a typical case, with an account of the autopsy. The patient was a man aged 39, and was a heavy drinker. He had been in hospital eight years previously with "alcoholic" neuritis and a very large liver. Ascites had begun ten months and œdema of the legs seven months before death. He was in hospital a month; he was tapped once, but the fluid rapidly reaccumulated. After tapping, the liver could be felt, but only two inches below the ribs. He was slightly jaundiced, and had melæna and hæmatemesis. Post mortem, there was a typical hobnailed liver weighing 59 oz., with some thickening of the capsule. The right heart was dilated. There were varices in the œsophagus, and evidence of gastritis. The peritoneum appeared slightly thickened. The spleen was enlarged, and there were parenchymatous changes in the kidneys. Saundby does not agree with those who consider that alcoholic cirrhosis is rather due to some other substance than ethylic alcohol which is present in alcoholic drinks, though admitting that pepper and capsicum may produce a certain amount of cirrhosis. He considers that ethylic alcohol is the primary predisposing cause, but that it may so act by favouring bacterial invasion of the liver from the intestine. In this he differs from those who, like Lanceraux, ascribe the damage to potassium salts used in the manufacture of wine, and those who, like Hale White, hold that the case against ethylic alcohol is not proven, and merely content themselves with "alcoholic drinks" as a causative factor in cirrhosis. Notwithstanding the fact that in this case a liver known to have been much enlarged seven years previously was found contracted and only a little above normal weight post-mortem, Saundby appears to hold that "cases of enlarged liver in which jaundice or pigmentation is an early and persistent symptom" belong to a different category. He goes on to point out that the effects of alcohol on the liver are often latent for long periods, and that minute inquiries are often necessary to elicit a history of alcoholic excess. The pain in the present case he ascribes to the gastritis and duodenitis, and the ascites to direct mechanical obstruction to the portal circulation. He advocates tapping the abdomen in all cases where the ascites causes inconvenience, and would repeat the operation, if necessary, unless rapid reaccumulation of the fluid shows that no permanent advantage can be gained. He does not apparently attach the grave prognostic importance to ascites apart from perihepatitis on which some authorities insist. He is in favour of the Talma-Morrison operation in suitable cases, but points out that it should not be undertaken as a last resort or as a routine treatment. He points out that the present case is exceptional to some extent in that severe hæmorrhage, and ascites co-existed; the fact that these two symptoms are not usually noted in the same case seems adverse to Saundby's view that they are both due to portal obstruction. Obstruction great enough to cause œsophageal varices would be naturally thought great enough to produce recognisable ascites. The spleen in Saundby's case was found, post-mortem, to be enlarged. Azzurini [2] has shown that the enlarged spleen in cirrhosis differs materially in its microscopical characters from the enlarged spleen of Banti's disease. In cirrhosis the changes are chiefly localised in the pulp, and are similar to those seen in stasis. Red corpuscles accumulate in the veins and cavernous tissue, causing venous dilatation, which may secondarily affect the Malpighian bodies, and there is much disintegration of the cells in the splenic pulp. In Banti's disease the primary change is in the Malpighian bodies, the reticulum is enlarged, but the veins are either not at all, or only slightly, distended.

**Cholelithiasis.**—Various substances have from time to time been advocated as solvents for gallstones, among which perhaps olive oil is the most widely known. In cases where operation is impossible, any drug which could reach the gallbladder and dissolve the stones to any extent would be an invaluable addition to our resources. Bain [3] has recently carried out some interesting experiments on dogs, with a view to determining the solvent action of certain drugs, and has also recorded clinical experiences which support the main results of his experiments. The latter may be divided into three series. In the first he merely placed gall-stones of known weight in the gall-bladder, and noted that they rapidly disappeared. In the second series he infected the gall-bladder with the bacillus coli when the stones were inserted, and found that the stones, though they lost some weight, were not dissolved in similar periods of time. In the third series the operation was similar to the second, but the animals were treated with sulphur, olive oil, chloroform, urotropin and iridin, cholelysin, calomel, or barium chloride. The last-named drug was given in too large doses, so that the result was equivocal. Of the rest, the only ones which apparently effected any solution of the stones were sulphur and the mixture of urotropin and iridin. Bain states that the old Harrogate sulphur water, combined with the *régime* of a health resort, is often successful clinically. He quotes much evidence to show that infection of the walls of the gall-bladder, with consequent catarrh, is the most important factor in the production and retention of gall-stones, and ascribes the effect of urotropin to its bactericidal action when excreted in the bile. This action has been demonstrated in a case of infection of the gall-bladder with the bacillus typhosus, where a biliary fistula existed. Bain considers that treatment of gall-stones by disinfection of the gall-bladder is rational, and likely to be successful in early cases where too much fibrosis of the walls and duct has not taken place. For early diagnosis he relies on a history of pain and dyspepsia, tenderness on deep pressure over the head of the gall-bladder, and in certain cases tenderness over the lower intercostal spaces behind. This symptom, if present in an acute attack, is also to be found during the quiescent intervals. These interesting and carefully reported observations certainly suggest a line of treatment which may be found useful, but it must be remembered that it is not always safe to argue from the results of experiments on animals, where the conditions may not be strictly similar to those obtaining in man. Dog's bile, for instance, differs in at least one important particular from human bile, the amount of taurocholic acid being much greater; and there is no direct evidence to show that gall-stones are as easily absorbed from the aseptic human gall-bladder as from that of the dog. Moreover, cases in man are reported where the symptoms have subsided after treatment with soap, olive oil, and other substances which Bain found to produce little or no effect on his experimental animals.

**Subphrenic Abscess.** — The various conditions which may give rise to a collection of pus below the diaphragm, and the various situations which such a collection may occupy, are pointed out by Bruce Clarke[4] in a clinical lecture. As he remarks, the term "subphrenic abcess" is only a convenient clinical expression for the terminal result of a large group of pathological processes. In the cases he describes the abcess was consequent on hydatid cyst of the liver, appendicitis (two cases), and gastric ulcer; and in one case the cause of two subphrenic abcesses, which appeared one after the other on either side of the spine, was not determined. Bruce Clarke points out that the diagnosis is rarely certain, interference with the action of the diaphragm, and rigors being the most usual signs. If left, most of these cases have a fatal termination, so that early surgical measures are necessary.

[1] Pract., June, 1905. [2] B. M. J. Epit., February 14, 1905. [3] B. M. J., August 5, 1905. [4] Clin. Jour., June 7, 1905.

